# Supplementary material for: Sexual dimorphism does not translate into foraging or trophic niche partitioning in Peruvian boobies (Sula variegata)
Source: PLoS One. 2025 Mar 18;20(3):e0320161. doi: 10.1371/journal.pone.0320161 (PMC11918401; doi:10.1371/journal.pone.0320161)
Supplement: S1 Table — Mean foraging trip metrics and at-sea behaviors ± standard deviations of incubating versus chick-rearing Peruvian boobies GPS tracked on Guañape Norte, Peru in 2016. For statistical tests, we used general linear mixed models specifying individual bird identity as a random effect, and with gamma distributions with log links for trip metrics and beta distributions with logit links for at-sea behaviors. No significant differences were found between groups for any metric. (DOCX) [file pone.0320161.s001.docx]

**Table S1. Foraging trip metrics of incubating versus chick-rearing Peruvian boobies.**

| **Metric** | **2016** | | **Test statistic** |
| --- | --- | --- | --- |
|  | **Incubating** | **Chick-rearing** |  |
| Individuals *(n)* | 4 | 6 |  |
| Total Trips | 4 | 11 |  |
| Trip Duration (hours) | 3.18 ± 2.5 (0.9–6.4) | 2.4 ± 2.0 (0.6–6.3) | *t* = 0.257, *p* = 0.797 |
| Trip Distance (km) | 112.0 ± 82.0 (30.6–199.0) | 89.7 ± 63.2 (24.8–207.0) | *t* = 0.144, *p* = 0.885 |
| Maximum Distance from Colony (km) | 44.0 ± 32.9 (11.8–74.4) | 36.4 ± 26.4 (7.2–86.5) | *t* = 0.081, *p* = 0.936 |
| Proportion Traveling (%) | 54.4 ± 27.6 (20.9–87.5) | 64.5 ± 25.1 (28.6–98.3) | *z* = -0.795, *p* = 0.427 |
| Proportion Resting (%) | 35.8 ± 23.3 (5.4–61.8) | 27.5 ± 26.2 (1.4–67.3) | *z* = 0.635, *p* = 0.525 |
| Proportion Foraging (%) | 9.9 ± 5.4 (4.8–17.3) | 8.0 ± 7.1 (0.3–26.3) | *z* = 1.015, *p* = 0.310 |

Mean foraging trip metrics and at-sea behaviors ± standard deviations of incubating versus chick-rearing Peruvian boobies GPS tracked on Guañape Norte, Peru in 2016. For statistical tests, we used general linear mixed models specifying individual bird identity as a random effect, and with gamma distributions with log links for trip metrics and beta distributions with logit links for at-sea behaviors. No significant differences were found between groups for any metric.
