# Supplementary material for: Sexual dimorphism does not translate into foraging or trophic niche partitioning in Peruvian boobies (Sula variegata)
Source: PLoS One. 2025 Mar 18;20(3):e0320161. doi: 10.1371/journal.pone.0320161 (PMC11918401; doi:10.1371/journal.pone.0320161)
Supplement: Attachment — Submitted filename: Response to Reviewers.docx [file pone.0320161.s002.docx]

RESPONSE TO REVIEWERS

*Wang et al. - PONE-D-24-10208*

**Associate Editor**

1. Please ensure that your manuscript meets PLOS ONE's style requirements, including those for file naming.
   - Response: We have revised the manuscript body and file names to follow the formatting laid out in the PLOS ONE style sheets.
2. When completing the data availability statement of the submission form, you indicated that you will make your data available on acceptance. We strongly recommend all authors decide on a data sharing plan before acceptance, as the process can be lengthy and hold up publication timelines. Please note that, though access restrictions are acceptable now, your entire data will need to be made freely accessible if your manuscript is accepted for publication.
   - Response: We are currently in the process of submitting our data to the Dryad repository and fully expect it to be submitted within the next few weeks in order to have it available before publication.
3. We note that Figure 1 in your submission contain [map/satellite] images which may be copyrighted. All PLOS content is published under the Creative Commons Attribution License (CC BY 4.0), which means that the manuscript, images, and Supporting Information files will be freely available online, and any third party is permitted to access, download, copy, distribute, and use these materials in any way, even commercially, with proper attribution. For these reasons, we cannot publish previously copyrighted maps or satellite images created using proprietary data, such as Google software (Google Maps, Street View, and Earth).
   - Response: The maps used in Figure 1 are provided by Stamen Design, which is fully open source under a Creative Commons Attribution (CC BY 4.0) license (see https://maps.stamen.com/#toner/12/37.7706/-122.3782). We have included an attribution statement in the Figure 1 caption.
4. Please include captions for your Supporting Information files at the end of your manuscript, and update any in-text citations to match accordingly.
   - Response: We have updated the manuscript to include the S1 Table caption already provided in the corresponding Supporting Information file.

**Reviewer #1**

The Peruvian booby (Sula variegata, booby hereafter) is a sexually dimorphic seabird living in a highly variable environment under the influence of El Niño Southern Oscillation (ENSO). The authors attempted to determine if there was niche partitioning between sexes by quantifying the foraging behavior and trophic niches of those boobies at Isla Guañape Norte, Peru, by comparing two years with different ENSO conditions. They expected fewer sex-based differences during a “normal” phase (2019) than during El Niño phase (2016, which was nonetheless rather weak). They found that although females were larger and in better body condition than males during both periods, this did not translate into foraging or dietary niche differences. They found that despite there being reverse sexual dimorphism (female larger than male), boobies did not show sex-specific foraging or isotopic niche partitioning and indeed had few differences in diet. The anchoveta pelagic fish (Engraulis ringens) predominated in both male and female diets independently of ENSO phase. They interpreted their results as indicating that there was little intraspecific competition between 2016 and 2019, not leading to the expected trophic niche differentiation.

I find it very valuable that “negative” results such as these are reported: It is –after all– exceptions that make “rules” interesting. Emphasis on confirmatory results is widespread, and for the reason expressed above, I find value in this sobering contribution.

That being said, I think that the exceptional nature of the results obtained from the years/ENSO phases contrasted is because the 2016 ENSO was really mild. I would have expected a more likely confirmation for the hypothesis tested if the comparison was made between a “normal” and a strong El Niño phase. Such study awaits to be conducted when the opportunity arises.

The paper is generally carefully written and well analyzed. I do not really have much to suggest about syntax and style. Indeed, the authors are extremely detailed in their presentation, which rather excessively lengthens their manuscript. But I prefer to have more than fewer details provided.

- Response: Thank you for your review of our manuscript. We have edited it taking into account your suggestions below: amending the list of keywords, providing more information on wing chord measurement, and amending Table 2.

More in a nit-picking mode, I may suggest:

- Keyword somewhat unnecessarily duplicate what is already in the title.
  - Response: We have amended the list of keywords to avoid duplication of content in the title.
- Table 1 and ff: On planet earth, one does not measure mass, only weight.
  - Response: Good point. There appears to be a lot of contention over this; see Murray (2008) and its response, Chardine (2008). Although the prevailing convention in the literature seems to report body mass over weight, Murray (2008) quite agrees with your original point. Chardine (2008) also makes a good point: when we weigh something to determine its mass (by proxy), we often use the resulting measurement to refer to the amount of matter in an object and not the force of gravity upon it. We have chosen to adhere to the common convention of reporting it as body mass.
    - *Chardine JW. Mass or weight: What is measured and what should be reported—Response to Murray (2007). The Auk. 2008 Jan;125(1):233–4.*
    - *Murray BG. Mass or weight: What is measured and what should be reported? The Auk. 2008 Jan;125(1):232–3.*
- And wing chord, was that flattened or naturally arched?
  - Response: A naturally arched wing chord measurement was taken. We have added this detail in the manuscript.
- Rather that SD, one should report 2SE, which immediately gives you an idea of the 95% confidence interval for the mean.
  - Response: While there are statistical and interpretive merits to reporting either metric (SD vs SE) for consistency, we have chosen to report SD as that is the metric most commonly reported by prior studies of Peruvian seabird morphometrics, movement and stable isotopes such as: Zavalaga et al. (2009). The Wilson Journal of Ornithology, 121(4), 739-744, Espinoza et al (2017) Marine Biology, 164, 1-15, and Zavalaga et al. (2010). Marine Ecology Progress Series, 404, 259-274.
- In Table 3: I cannot find the asterisks announced in the legend.
  - Response: Having looked over the paper, we found that Table 2 is the table with missing asterisks. However, we have revised it to leave the significant pairs of comparisons reported only in the text. We have also corrected a decimal error in the numbers for 95% and 50% kernel area estimates both in Table 2 and in the relevant text.

**Reviewer #2**

Sexual dimorphism does not translate to foraging or trophic niche partitioning in Peruvian boobies (Sula variegata)

Overall appreciation

This manuscript assessed the sexual and interannual differences (between two years) on the foraging distribution, at-sea behaviour, isotopic niche (δ13C-δ15N), and diet composition (through regurgitate analysis) of Peruvian boobies, during a year of neutral vs. weak El Niño conditions. Overall, the manuscript is well-written and provides new insights into the ecology of Peruvian boobies during the breeding season. However, I found some parts unclear throughout the manuscript. My comments offer suggestions for improvement, but in other cases I raise questions or suggest additional justification is needed. I hope my comments are clear, potentially revealing aspects of the work that I may have misunderstood and are helpful for improving the manuscript.

- Response: We appreciate the time you put into this very thorough, comprehensive, and constructive review. We have revised the manuscript to take into account your suggestions and advice. The line numbers noted below refer to the revised manuscript with track changes. Many thanks!

Major concerns:

- The premise that weak El Niño conditions would trigger differences in foraging and trophic ecology lacks sufficient support from the literature. I believe this premise is not strong enough to justify assessing interannual differences.
- Response: We revised our manuscript to better clarify and justify our chosen approach and premise. Specifically, we chose to assess interannual differences in addition to sex differences based on a number of factors in combination that support low prey availability (known to affect niche partitioning in seabirds, see Paiva et al. 2017 for an example) during the weak EN of 2016:
  1. A very strong EN preceding sampling and affecting the birds in that year.
  2. Clear differences in the nesting status of the booby colony (much lower breeding numbers in 2016) that suggest a decline in prey availability. We have reported this and the preceding ENSO conditions in the manuscript (Line 100-112).
  3. In addition, in the same Humboldt Current System, we can see an effect of ENSO condition (and subsequent resource availability) on potential foraging behavior, as in the effect of sex*SSTa on differing isotopic niches of male and female Peruvian fur seals (Edwards et al. 2021). Both papers are now cited in the manuscript.
     a. *Edwards MR, Cárdenas-Alayza S, Adkesson MJ, Daniels-Abdulahad M, Hirons AC. Peruvian Fur Seals as Archivists of El Niño Southern Oscillation Effects. Front Mar Sci. 2021 Nov 15;8:651212.*
     b. *Paiva VH, Pereira J, Ceia FR, Ramos JA. Environmentally driven sexual segregation in a marine top predator. Sci Rep. 2017 Jun 1;7(1):2590.*
- The lower sample size in 2016 (n = 10) compared to 2019 (n = 32) adds uncertainty to the interannual assessment, especially when comparing sexes within each year (7 females vs. 3 males in 2016; 20 females vs. 12 males in 2019). There are also some misconceptions in the methodology and some biased interpretations of the results.
  - Response: The differences in sample size are a result of the conditions in each year. In revision, we have provided additional clarity as to why the sample size was so low in 2016 and why data from this year may be more representative than apparent. Specifically, we clarify the low breeding propensity of birds in this poor year and how it provides insights into the species ecology during a unique period when conditions are poor enough to reduce but not completely prohibit breeding. Regardless, in our revised manuscript we have sought to address the above comments, clarify uncertainty due to the group sizes, and interpret results within appropriate boundaries (see our specific responses to relevant comments below for where we have updated the manuscript and added caveats to address these concern).
- I strongly recommend the authors revisit some crucial aspects of the statistical design, particularly in the analysis of GPS data and regurgitate data, as well as other aspects related to tracking data representativeness and calculation of smoothing parameters.
  - Response: Thank you for the very thorough feedback regarding this portion of the manuscript. We have gone through and addressed each one (responses to your comments regarding Lines 192-193, 209, 214-215, 216-217, 218, 220 for GPS; 236-240 for regurgitates) below, and revised the methods and/or emphasized the caveats in the manuscript in response.
- I am not entirely convinced of the importance of analysing and discussing the degree of sexual dimorphism in this study. In fact, there is no linear pattern found for this topic. Instead, I would expect a discussion focused on the impact or influence of sexual dimorphism on adult foraging metrics, at-sea behaviour and spatial segregation, diet composition, and isotopic niche, rather than just a comparison with the degree of sexual dimorphism in other species. While it is a good premise to discuss your results, it should not be discussed exhaustively.
  - Response: In response we have cut down considerably the discussion of degree of sexual dimorphism to allow more focus on the other parts of the discussion. The influence of sexual dimorphism on sexual and interannual differences are integrated in each respective section, and we believe that now the section on sexual dimorphism appropriately frames the rest of the discussion in a helpful way.
- The discussion is not structured in an easily understandable way. I suggest dividing the discussion into two sections: one for sexual differences and another for interannual differences (even though I have significant doubts about the representativeness of GPS and isotopic data in 2016). This should simplify the discussion and avoid the use of many subsections that, in my opinion, do not warrant such emphasis.
  - Response: We appreciate this suggestion; however, after consideration we have opted to retain the overarching structure (which at the highest level does line up with sexual and interannual differences) of the discussion. We have also kept the subheadings. As we tested many variables and used multiple methods in this paper, we feel the mirroring of the discussion structure to that of the results, as well as an introductory overview of the main findings, helps to keep things organized. It is not necessarily a thematic device, but more of an organizational one. In addition, we believe that the paring down of the discussion on sexual dimorphism (to convert it into more of a framing device) now makes the discussion clearer and more easily understandable.

Specific comments

Title

- Amend “translate to foraging” to “translate into foraging”.
  - Response: Revised as suggested.

Abstract

- Line 2: amend “marine birds” to “seabirds”.
  - Response: Revised as suggested.
- Line 14: I would amend to ‘sexual dimorphism’, removing the ‘reverse’. If the males were larger than the females these would be expected anyway.
  - Response: A good point towards conciseness; revised as suggested.
- Line 19-20: And when they are unfavourable too, correct? According to the results, authors have stated there were no interannual differences, so I would rather suggest that oceanographic conditions were not that strong to influence prey intake, or the type of prey captured by males and/or females in both years. In fact, it suggests that both prey and predators are adapted to these conditions in the Humboldt Current System.
  - Response: To address this comment we have revised the wording regarding favorable conditions. However, we have also included information about the differences in ENSO and nesting successes of the colony during the sampled years in previous lines, and added a clause pointing out the effect of potential prey availability and oceanographic conditions on the breeding individual vs. the population as a whole.
- Keywords: The use of “niche partitioning”, “Peruvian booby”, and “sexual dimorphism” as keywords will not increase the range of readers since they are already mentioned in the title. Try to use different keywords, like: Sulidae, resource partitioning, reverse sexual dimorphism (RSD).
  - Response: We have removed keywords and replaced them as suggested.

Introduction

- Line 12-13: I suggest changing to “sexual dimorphism” or “size-related sexual differences”.
  - Response: Revised as suggested.
- Line 62-63: This sentence is redundant. In the two previous sentences authors have already reported the “slight” differences on the foraging metrics.
  - Response: We have removed the sentence and revised the following one for smoother flow.
- Line 66: The references are not properly formatted.
  - Response: Thank you; we have corrected the citations.
- Line 72-73: I would say this is not an expectation concerning the already known reverse sexual dimorphism. What authors could expect is a higher or lower size dimorphism index (SDI).
  - Response: A good suggestion to refine and clarify what we mean. We have revised as suggested.
- Line 76-79: I fully agree with this, however it felt incomplete. I would suggest attributing specific expected patterns to males and females regarding foraging metrics/characteristics, behaviour, isotopic niche, prey intake, mass, or size. Despite previous studies reporting low evidence of sex-based differences in tropical environments, authors can infer expected differences in this study, particularly under less favourable conditions. Indeed, I would expect a priori a competitive advantage for females due to their larger body size. For instance, Almeida et al., 2021 (already cited in this manuscript) observed sexual segregation in brown boobies only during a period of co-existence with another breeding sulid, indicating a period of higher adult density within the foraging range.
  - Response: Good suggestions. To keep things succinct, we have added a sentence mentioning the potential differing response (and advantage) of females versus males due to their body size.

Materials and Methods

- Line 105: Chicks or eggs.
  - Response: Revised as suggested.
- Line 109: The reference is not properly formatted.
  - Response: We have corrected the citation.
- Line 116: How were the feathers marked?
  - Response: We marked feathers on the head using a PAINTSTIK livestock marker (LA-CO Industries). We have added this information into the manuscript.
- Line 124-125: Perhaps I have missed something, but it looks like the tag was only deployed on the adults for a few hours.
  - Response: There are always trade-offs when deploying tags. We chose to deploy tags for shorter periods of time for several reasons. The first was to be able gather more data from different individuals in order to minimize pseudoreplication. Secondly, the device battery life was fairly short since we worked with high-resolution data loggers (which record ~1 location/second). And lastly, we aimed for short deployment times in order to minimize any possible handicapping effect the tags had on the birds.
- Line 130-132: If I am not wrong, the blood cells are those who have a turnover rate of 28 days for δ13C and δ15N values. The turnover rate in whole blood is larger than that, even though comparable isotopically speaking according to Cherel et al. (2005).

Cherel, Y., Hobson, K.A., Bailleul, F., Groscolas, R., 2005. Nutrition, physiology, and stable isotopes: new information from fasting and molting penguins. Ecology 86, 2881–2888.

- Response: Please refer to Barquete et al. (2013) and Bearhop et al. (2002), especially the figures in each of these papers. Plasma has the fastest turnover rate, red blood cells have the slowest, and whole blood is somewhere in between. From Barquete et al. 2013, “For dietary reconstruction using stable isotope ratios, our findings suggest that blood plasma provides dietary information integrated over a period of ~ 15 days, total blood of ~20 days and erythrocytes over a month.”
  - *Barquete V, Strauss V, Ryan PG. Stable isotope turnover in blood and claws: A case study in captive African Penguins. Journal of Experimental Marine Biology and Ecology. 2013 Oct;448:121–7.*
  - *Bearhop S, Waldron S, Votier SC, Furness RW. Factors That Influence Assimilation Rates and Fractionation of Nitrogen and Carbon Stable Isotopes in Avian Blood and Feathers. Physiological and Biochemical Zoology. 2002 Sep;75(5):451–8.*
- Line 154-156: I would rather suggest the use of a radius (perhaps of 200 m). It is more uniform.
  - Response: We agree, and we actually did use a radius when processing the movement data though it was not clearly noted in our initial submission. We have revised the manuscript here so that our process is clearer.
- Line 159-161: This is why I prefer to use a radius. Sometimes, boobies can interact when at the colony or when at the arriving/landing which may disturb each other and result in brief flights. According to the authors’ data filtering method, the occurrence of false foraging trips seems likely.
  - Response: Thank you for the helpful feedback. We have clarified our methods in regards to the GPS data processing (see above response). Having also gone through and visually confirmed the tracks for each individual, we are confident that each foraging trip is a legitimate trip and that all non-foraging activity has been removed.
- Line 184-185: Perhaps some literature support in here or at least a brief explanation of why pooling these two categories together.
  - Response: According to Garriga et al. 2016, “A partition with only three clusters (LL,LH, and HL), with the HH cluster absorbed partly by the LH and partly by the HL clusters, would probably represent a better behavioural classification. However, the likelihood pay-off of this solution prevents the algorithm to reach it.” For multiple reasons, we initially suspected that it was likely in this dataset that many of the HH (relocating) points were more likely to be grouped in with HL (traveling). However, we have revisited the way we assigned the behavior groups and decided to ungroup the HL and HH categories for transparency and robustness. Therefore we re-ran the analysis with separate groups (the results did not change significantly). We have revised the methods, results, and discussion sections accordingly (Line 209-210, 238-245, 249, 387-389, 545-546, Table 2).
- Line 192-193: The smoothing parameter was calculated for each group? If yes, 3 males in 2016 is a very low number of individuals to compute such parameter. Please clarify.
  - Response: Yes, this is correct. We have edited the text to make this clear. Given the high frequency of fixes (once every 2-10 seconds), we had a relatively large dataset for the group, well above the reported thresholds of locations per animal at which level of relative bias was acceptable (several figures cited: 200 per animal; minimum of 30 or preferably > 50; 40 locations/month for GPS data). We decided to use the *ad hoc* smoothing method as it is found to perform better over a range of sample sizes in terms of relative bias.
    - *Schuler KL, Schroeder GM, Jenks JA, Kie JG. Ad hoc smoothing parameter performance in kernel estimates of GPS‐derived home ranges. Wildlife Biology. 2014 Oct;20(5):259–66.*
- Line 209: Aside from all these metrics, an important request when working with tracking data at the population level is the representativeness of the data. Have authors checked for it? A good way to verify the representativeness of the foraging areas is to follow the method described here: Lascelles, BG et al. Applying global criteria to tracking data to define important areas for marine conservation. Divers Distrib 22: 422−431 (2016), and applied here: Cecere, JG et al. Spatial segregation of home ranges between neighbouring colonies in a diurnal raptor. Sci Rep 8: 11762 (2018); here: Weimerskirch, H. et al. At-sea movements of wedge-tailed shearwaters during and outside the breeding season from four colonies in New Caledonia. Mar. Ecol. Prog. Ser. 663, 225–238 (2020); and here: dos Santos, I. et al. Sexual segregation in the foraging distribution, behaviour, and trophic niche of the endemic Boyd’s shearwater (Puffinus lherminieri boydi). Mar Biol 169 (2022).
  - Response: Thank you for these comments and suggestions. In response, we have revised our manuscript to better clarify in what ways our data is representative of the population each year and provide caveats to the strengths and weaknesses of our datasets. For example, in our revised methods we provide a proxy of breeding activity in each year and note the very low breeding numbers in 2016. We show how the 10 individuals tracked in 2016 represented 6.7% and 15.6% of breeding males and females in our study plot, whereas the 32 individuals sampled in 2019 represented 1.65% and 2.7% of breeding males and females in the same plot, respectively. This highlights how our data from 2016 is likely more representative of the overall breeding population than may be immediately apparent based on sample numbers alone. In addition, we used a null-hypothesis randomization approach when assessing potential significant spatial overlaps or the lack thereof (see Table 3) which provides added assurances of our results given the low sample numbers and uneven group sizes. However, we agree that our data is shy of the 25-50 trips per group recommended by Lascelles et al. (2016) to achieve representativeness of the population. While our added context on breeding numbers and proportions are helpful, we do agree on the challenges inherent to low sample sizes. As such, we have added considerable additional context and caveats in both the methods (Line 130-138) and the discussion (Line 567-580).
- Line 214-215: Have authors tested for multicollinearity? If not, it should be done because it is not necessary to test collinear variables.
  - Response: We are not concerned about multicollinearity per se because we are not using these multiple variables as predictors in a single regression model. More to the point, we ran separate tests on multiple response variables that may be related. However, you raise a valid concern given the potential for the response variables in these separate tests to be correlated. We therefore tested for relationships among each of the response variables and have now reported on the correlations between variables in the methods (Line 237-245, 268-271). We have also updated some of our statistical design to account for these correlation matrices (i.e., reduce/remove “redundant” analyses of strongly correlated response variables) and changed the reported results accordingly (Line 373-375, 387-389).
- Line 216-217: My only concern with this design is the lack of males successfully tracked in 2016. I truly believe that this may have influenced the results.
  - Response: Indeed. Unfortunately, there were not many males we were able to capture and track in 2016 as there were very few birds (of either sex) breeding. We have added more detailed caveats to the methods, discussion, and conclusion (as mentioned above in our response to your comment regarding Line 209).
- Line 218: Have authors checked the residual deviance of the random factor, the residuals of the model through qqplots (normality) and homogeneity of variances (to detect potential biases)?
  - Response: Yes. We checked the residuals for the trip duration model as well as the random effect (individual bird) and both qqplots for normality and homogeneity of variance are satisfactory. We have included this information in the text for clarity (Line 252). If by residual deviance of the random factor you mean comparing model fits, a model without the random factor returned slightly lower AIC (41.9 vs 43.9), but we chose to include the random effect in the model due to the biological relevance of variability between individuals.
- Line 219: Were these data transformed? I am asking because authors have mentioned the log-transformation of the foraging metrics but not the behaviours. Please clarify.
  - Response: As the at-sea behaviors are proportional (and bounded by 0 and 1), we did not transform them and instead used a binomial distribution when they were fitted to a model. We have clarified this in the text (Line 254).
- Line 220: Any p-adjustments for multiple modelling for the using of the same dataset?
  - Response: We have updated the tests we performed to include only proportions of time spent foraging and relocating, which were not correlated. Due to the relatively low number of tests used (2), the lack of correlation between response variables used in each individual test , and the low probability of Type I error, we have not used any p-adjustments for these models.
- Line 231: What does this mean precisely? Percent of prey by mass of regurgitate? Please clarify.
  - Response: We have amended the text to clarify. We quantified the species composition of each regurgitate by percent mass.
- Line 236-240: My concern here is that some of these metrics may be correlated as in foraging trip parameters. For instance, higher the number of prey items, higher the total regurgitate mass; or higher the individual prey size, lower the number of prey items. So, these tests are not independent, and this should be considered.
  - Response: A good suggestion. We have used a Spearman rank correlation to test correlations between these variables (as also mentioned above in response to your comment regarding Line 214-215) and given the results in this case have chosen to retain all the variables. We have revised the methods to include the correlation test (Line 268-271).
- Line 251: Since no context was given about stable isotopes and their usefulness in ecology and food web studies, it would be informative to add in here a brief explanation of how carbon and nitrogen can be informative in diet studies.
  - Response: We have edited and added in the isotopic niche analysis methods a clearer, brief explanation of what carbon and nitrogen can indicate about the resources a consumer is using (Line 302-305).
- Line 253: Just a minor correction, the only unit that does not need a space between the number and the unit is the percentage (%). For all the other units authors should use the space between.
  - Response: Interesting! Although it seems that convention has shifted and most recent articles do not include a space, the traditional recommendation is indeed to include it. We have checked and updated the entire manuscript accordingly.

Results

- Line 310: Two decimal cases are sufficient for the tests and three decimal cases for the p-values.
  - Response: We have corrected all test statistics on this line and in the rest of the results so they report to two decimal places (or three, in the case of very small values).
- Line 322-326: What is the reason for testing annual differences on morphometrics? With exception of body mass that could indicate a lower overall condition, it would already be expected that females were larger than males.
  - Response: We did this to ensure there were no incidental differences (i.e. groups are similar between years). If differences had been found, that would be a consideration and/or caveat when interpreting foraging/dietary results later on.
- Line 334-340: I would resume this section in: “There were no annual either sex differences on any of the foraging trip metrics (minimum F < F < maximum F; p-value > minimum p-value). Also, as mentioned before, these variables are probably collinear. If so, authors only need to test the effect of one out of three predictors.
  - Response: Good suggestion. Based on a correlation matrix, we have revised our statistical design to only test trip duration, as we mentioned above in response to your comment regarding Line 214-215.
- Line 344-347: I am not aware of the journal limits of characters in the legends of tables and figures, but if it is possible, include this part in the main legend as well. The same for the other tables.
  - Response: Apologies if these lines were formatted unclearly. This section is the legend for Table 2, not part of the manuscript body. All tables are currently formatted according to PLOS ONE’s guidelines.
- Line 349: Testing all these proportions, authors could not guarantee the independence of data. These variables are highly dependent from each other since they correspond to the proportion of time spent in each one of the four categories. I would rather test the foraging behaviour.
  - Response: As guessed, our main interest was indeed to check for differences in the “foraging” proportions. To address this comment, we tested the correlations between the four variables (traveling and relocating groups have been split, as mentioned above) and based on these relationships, we proceeded to only analyze proportion of time spent foraging and proportion of time spent relocating. We have updated the results accordingly (Line 387-389).
- Line 425: Is there a reason for table 3 comes after table 4 in the manuscript?
  - Response: Good catch. We have reversed the numbering of the tables so they are in numerical order.
- Line 426: Just a minor correction, the delta sign (as alpha, beta, gamma) must be in italics.
  - Response: We have italicized all instances of the delta sign in the manuscript.
- Line 430: Just a minor correction, the symbol used for carbon values is not a minus sign as in maths, but a “–“.
  - Response: We have gone through the manuscript and replaced the hyphen-minus usage in the stable isotope portion with minus symbols (Unicode U+2212). We have also amended the usage of a hyphen-minus instead of a minus when appropriate in other sections of the paper.
- Line 451-460: No need to present both sexes in relation to one another. Just present females realtive to males or males relative to females. Otherwise, it is redundant.
  - Response: As the *nicheROVER* analysis yields directional pairwise overlap values, it is important to report both overlap values for each comparison between two groups – niche space overlap may not be symmetrical for the groups being compared.
- Figure 3: This figure needs considerable improvements. The legend of the figure is overlayed with the fourth plot. The symbol per mil could be represented instead of “per mil” in the xx’ axis title. The use of more contrasting colours and the use of a colour-blind friendly pallette are recommended.
  - Response: Thank you for the feedback. We have revised the figure considerably. The axes lengths are now proportional to each other, the legend has been relocated, and we have replaced the “per mil” with the symbol. The colors have also been changed to be more colorblind-friendly while also matching the color coding for our other figures.

Discussion

- Line 469-470: I would remove this sentence since this was not a main finding of your study. This was already reported.
  - Response: Good point. We have cut the sentences considerably and revised them for succinctness.
- Linbe 476-477: This was one of the most interesting results and it was not given much relevance.
  - Response: Yes, we agree! While we have a paragraph in the discussion that details this (Line 591-602), we have added a sentence into the conclusion (Line 772-774) to emphasize this finding.
- Line 479: “both sexes spent proportionally less time foraging in 2019 than in 2016.” This section looks disconnected from the sentence. Perhaps starting a new sentence when shifting the subject to foraging activity.
  - Response: Revised as suggested.
- Line 483-511: This would be useful on explaining the differences, or in this case the absence of differences, on foraging metrics, behaviour, and isotopic niche instead of describing something that was already known amnd compare with other populations or even species. Do not get me wrong, it is a good premise to discuss your results but not to be described exhaustively.
  - Response: We have edited this section and reduced it considerably. We have also included some brief sentences on the relation or lack thereof of the sexual dimorphism on the foraging/diet/isotope metrics we examined. We leave more extensive discussion of sexual and interannual differences in each of their respective sections.
- Line 561: The reference is not properly formatted.
  - Response: We have corrected the formatting.
- Line 573: Authors can also add the ref 52 (Almeida et al. 2021) in red-footed and brown boobies.
  - Response: We have added additional information regarding sex differences in δ^13^C values in BRBO and cited the paper.
- Line 584-587: Also, it is possible that: 1) El Niño weak conditions in 2016 were not enough (similar prey abundance and availability to adults) to trigger sexual segregation on diet composition and/or on foraging behaviour while at sea; 2) The low sample size in 2016 could have masked some potential sex-related differences on isotopic niches.
  - Response: Good points. We have added these possibilities suggested above into the discussion (Line 567-569, 625-631).
- Line 597-598: It could have extended their foraging ranges, travel distances, and trip duration. Interestingly, this pattern was only detected for females, at least concerning the home range areas (95% KUD).
  - Response: Yes, it is quite interesting. As noted in the manuscript, we found mixed support for this concept as while females’ kernel areas differed noticeably between years, other metrics of foraging such as trip duration did not.
- Line 614-616: Out of curiosity, are there some strong evidence in the literature of different parental roles in this species?
  - Response: In fact, there are some older papers (by Nelson, cited in the manuscript) on the differing parental roles in Peruvian boobies. Males tended to brood more and were noted to “specialize in inshore fishing in shallow water”, while females contributed more to provisioning, especially when chicks are older. We have added this information and the citations in this section (Line 673-676).
- Line 618-620: Indeed this can happen. However, with weak El Niño conditions it would not be highly expected. Moreover, the prey mass did not change from 2016 to 2019, it has only varied between sexes, mostly due (I would say) to size-related sex differences.
  - Response: You make very good points. In our case, the weak El Niño at the time of sampling was preceded by a very strong El Niño which gradually declined into a prolonged moderate-to-weak EN that lasted most of the calendar year 2016 (February to July). We contextualized this particular event in the methods (Line 100-103). Birds were experiencing poor conditions up to and after our sampling period and had a low breeding propensity. It may be measuring only breeding adults may have selected for, of course, adults that were able to meet the minimum energetic requirements/foraging success to breed (of which prey mass could be one indicator). On a population level, boobies were less successful in 2016 than in 2019 (when looking at numbers of breeding birds). On an individual level, when only sampling those adults who opted into breeding, it makes sense that their foraging and diet metrics would be similar between years – otherwise in this species particularly reliant on anchoveta availability for chick-rearing, adults would choose to forgo breeding. We have also added this context into various places in the discussion section (Line 505-510, 523-526, 688-692).
- Line 623: Please amend “dispersed” to “sparse”.
  - Response: We have revised as suggested.
- Line 623-624: What is the difference between “laterally” and “horizontally”. Please clarify.
  - Response: Good catch. We have revised to “vertically and horizontally”.
- Line 626: Please amend “labor” to “parental duties” or just “duties”.
  - Response: We have revised as suggested.
- Line 627: Please amend “prey scarce years” to “years of lower prey availability” or “years of prey scarcity”.
  - Response: We have revised as suggested.
- Line 638: Too much ‘howevers’. Avoid the over use of adverbs.
  - Response: Agreed. We have corrected the repetition in the paragraph.
- Line 675-677: Peruvian boobies are quite specialist then. This scenario is similar to the extreme breedigng failure of common guillemot on the coast of Britain in 2004, which was due to the demographic collapse of sandeels (Wanless et al., 2005).

Wanless, S., Harris, M. P., Redman, P., & Speakman, J. R. (2005). Low energy values of fish as a probable cause of a major seabird breeding failure in the North Sea. Marine Ecology Progress Series, 294, 1-8.

- Response: Interestingly enough, there is a latitudinal gradient in the degree of specialization in Peruvian boobies, so the answer is – it depends where you are! As mentioned in the paper, northern populations have almost 100% of their diet composed of anchoveta, and these are the ones that in anchoveta-poor years are more likely to desert the islands and forgo breeding altogether. Southern boobies are less likely to – in Chile they have a much more mixed diet (perhaps 0-50% anchoveta, based on conditions).
- Line 713: The shift of isotopic baselines of carbon and nitrogen is not the only factor to mention here. In fact, the absence of support of the different approaches used in this study could be due to three factors (in separate or in combination):

1) The individuals sampled for diet, isotopes analysis, and those tracked with GPS were not the same. Thus, inter-individual differences were not possible to take into consideration;
2) The representativeness of this Peruvian’s booby population was not guaranteed by the number of individuals tracked with GPS devices;
3) the large differences between the sample sizes of 2016 and 2019 may have also biased the results, hiding or not possible interannual patterns.

- Response: These are all good points. We have added these caveats in the beginning of the discussion where we introduce our findings (Line 514-517), as well as added them into the discussion subsection where they are relevant (Line 567-580, 688-692, 764).
