# Supplementary material for: Sexual dimorphism does not translate into foraging or trophic niche partitioning in Peruvian boobies (Sula variegata)
Source: PLoS One. 2025 Mar 18;20(3):e0320161. doi: 10.1371/journal.pone.0320161 (PMC11918401; doi:10.1371/journal.pone.0320161)
Supplement: Attachment — Submitted filename: Wang et al_comments revised version.docx [file pone.0320161.s003.docx]

Sexual dimorphism does not translate into foraging or trophic niche partitioning in Peruvian boobies (*Sula variegata*)

General comment:

Overall, I am truly satisfied with this revised version of the manuscript entitled “Sexual dimorphism does not translate into foraging or trophic niche partitioning in Peruvian boobies (*Sula variegata*)”. The authors have responded fully to the comments made during the review, and I commend the authors for completing such an extensive revision. The changes made in response to the reviewers' comments have significantly improved the quality of the manuscript, which is now almost ready for publication. I have only a few minor comments to make before acceptance.

Minor comments:

Line 100-112: I appreciate this clarification. Now it is much more perceptible that the proportion of the nests tracked during this study was relevant in both years.

Line 279: Remove the comma in “homogeneity of variance, and is”.

Line 552: Amend “among” to “between”.

Line 569: Remove the “of” in “asymptotic relationship of between”.

Line 575: Remove the duplicate “in”.

Line 586: Suggest remove “also”.

Line 586: Suggest remove “than males did on average”. It is redundant since you are always comparing males and females.

Line 589: Remove “for us”.

Line 593: Replace “or” by “nor”.

Line 596: Amend “mass” to “size”.

Line 609: Suggest remove “we observed”.

Line 738: “tissue *δ*^13^C value”. No need for “tissue” in here.

Line 765: Remove “us”.

Line 765: Remove “and studies”. No need for it I guess.

Line 775-776: “there was some evidence to suggest”. No need to be that cautious here. I would remove “to suggest” from the sentence.

Line 777: I think “present in 2016” could be removed. Authors have already categorised the years of respective weak El Niño and La Niña years above.

Line 778: Remove “in each year”. Anchoveta was abundant across the years.

Line 781: “Interannual variation in prey abundance can be extremely high in the HCS (23) and so it is possible foraging niche segregation between sexes may occur in response during years of lower prey availability than what was observed in our study.” I would divide in two separate sentences. Like, “Interannual variation in prey abundance can be extremely high in the HCS (23). Thus, sex-related foraging niche segregation may occur during years of lower prey availability than what was observed in our study.”
